# Notch Signaling Regulates MMP-13 Expression via Runx2 in Chondrocytes

**DOI:** 10.1038/s41598-019-52125-5

**Published:** 2019-10-30

**Authors:** Di Xiao, Ruiye Bi, Xianwen Liu, Jie Mei, Nan Jiang, Songsong Zhu

**Affiliations:** 1 State Key Laboratory of Oral Diseases & National Clinical Research Center for Oral Diseases & Department of Orthognathic and TMJ Surgery, West China Hospital of Stomatology, Sichuan University, Chengdu, China; 2 Department of Stomatology, The First Affiliated Hospital of Dalian Medical University, Dalian, Liaoning China; 3 Department of Oral and Maxillofacial Surgery, Guangdong Provincial Hospital of Stomatology, Southern Medical University, Guangzhou, China

**Keywords:** Single-molecule biophysics, Transcriptional regulatory elements

## Abstract

Notch signaling is involved in the early onset of osteoarthritis. The aim of this study was to investigate the role of Notch signaling changes during proliferation and differentiation of chondrocyte, and to testify the mechanism of MMP-13 regulation by Notch and Runx2 expression changes during osteoarthritis. In this study, Chondrocytes were isolated from rat knee cartilages. Notch signaling was activated/inhibited by Jagged-1/DAPT. Proliferative capacity of Chondrocytes was analyzed by CCK-8 staining and EdU labeling. ColX, Runx2 and MMP-13 expressions were analyzed as cell differentiation makers. Then, Runx2 gene expression was interfered using lentivirus transfection (RNAi) and was over-expressed by plasmids transfected siRNA in chondrocytes, and MMP-13 expression was analyzed after Jagged-1/DAPT treatment. *In vivo*, an intra-articular injection of shRunx2 lentivirus followed with Jagged1/DAPT treatments was performed in rats. MMP-13 expression in articular cartilage was detected by immunohistochemistry. Finally, MMP-13 expression changes were analyzed in chondrocytes under IL-1β stimulation. Our findings showed that, CCK-8 staining and EdU labeling revealed suppression of cell proliferation by Notch signaling activation after Jagged-1 treatment in chondrocytes. Promoted differentiation was also observed, characterized by increased expressions of Col X, MMP-13 and Runx2. Meanwhile, Sox9, aggrecan and Col II expressions were down-regulated. The opposite results were observed in Notch signaling inhibited cells by DAPT treatment. In addition, Runx2 RNAi significantly attenuated the ‘regulatory sensitivity’ of Notch signaling on MMP-13 expression both *in vitro* and *in vivo*. However, we found there wasn’t significant changes of this ‘regulatory sensitivity’ of Notch signaling after Runx2 over-expression. Under IL-1β circumstance, MMP-13 expression could be reduced by both DAPT treatment and Runx2 RNAi, while Runx2 interference also attenuated the ‘regulatory sensitivity’ of Notch in MMP-13 under IL-1β stimulation. In conclusion, Notch signaling is an important regulator on rat chondrocyte proliferation and differentiation, and this regulatory effect was partially mediated by proper Runx2 expression under both normal and IL-1β circumstances. In the meanwhile, DAPT treatment could effectively suppress expression of MMP-13 stimulated by IL-1 β.

## Introduction

Osteoarthritis (OA) is the most common degenerative articular disorder nowadays^1^. Currently, there haven’t been effective interventions to prevent the initial onset or arrest the progression of OA, which is due to the limited understanding of molecular mechanisms in the progress of the disease.

Healthy articular cartilage consists of chondrocytes and extracellular matrix (ECM). Under the physiological condition, chondrocytes produce both catabolic and anabolic proteins to keep the equilibrium of ECM, and ECM in turn functions to maintain the homeostasis of cellular environment and the structure of cartilage^2^. Once OA is initiated, the tightly controlled metabolic balance of ECM will be disrupted by expression changes of certain genes in chondrocytes, leading to progressive loss of matrix proteins. Fundamentally, cleavage of Col II is considered as a critical early event of OA^2–4^.

Initial cleavage of Col II is preferentially performed by Matrix metalloproteinase 13 (MMP-13), an interstitial collagenase expressed by articular chondrocytes^5–7^. MMP-13 expression could be extraordinarily detected in the vicinity of early focal lesion in OA cartilage^8^. Therefore, characterizing the regulation of MMP-13 expression in chondrocytes is important to understanding molecular mechanisms of early OA.

Runx2, which is known with a unique binding site in the MMP-13 promoter region, might be a focal point of abnormal signaling mechanisms that promote OA initiation^9^. In Runx2-haploinsufficient (Runx2^+/−^) mice, reduced MMP-13 was observed, and the pathology of instability-induced experimental OA was ameliorated by decreasing cartilage destruction^10^. Evidences above illustrate that Runx2 contributes substantially to pathogenesis of OA disease through its regulatory links to MMP-13.

Similarly, MMP-13 expression could also be stimulated by Notch signaling pathway in murine articular chondrocytes and rat OA model^11,12^. Notch signaling is an evolutionarily conserved pathway that mediates cell-cell interaction required for cell fate determination^13^. This signaling is activated when a ligand-receptor interaction induces a series of receptor proteolytic cleavage to release Notch1 receptor intracellular domain (Notch1-IC or Notch1-ICD, NICD) in cytoplasm. The NICD then translocated into nuclei and ultimately induces expression of downstream target genes^14^. Recent reports raised the issue of involvement of Notch signaling in the homeostasis of articular cartilage and pathogenesis of OA process^15–17^. Loss of RBPjκ-dependent Notch signaling in postnatal joint cartilage results in an early OA-like pathology^18^, while temporary suppression of Notch signaling in murine joints leads to delayed OA progression^19^. These data collectively indicate that there is a requisite role of Notch signaling in the onset and progression of OA. In addition, Shang *et al*.^20^ recently found that there is a significantly positive correlation between Runx2 expression, as well as MMP-13 expression, with Notch signaling in a chondrogenic cell line. All these studies above led us to hypothesize that MMP-13 expression was possibly regulated by Notch signaling through activation of Runx2 in chondrocytes, which regulatory mechanism might be required for the pathogenesis of early OA.

To test our hypothesis, we imitated alteration of Notch signaling in chondrocytes using exogenous recombinant rat Jagged-1 protein (as Notch signaling ligand) and N-[N-(3,5-Difluorophenacetyl)-L-alanyl]-S-phenylglycine t-butyl ester (DAPT) (as Notch signaling γ-secretase inhibitor) respectively. Then proliferation and differentiation of mature chondrocytes were examined. Furthermore, Runx2 transcription was interfered by shRNA in Jagged-1/DAPT treated cells and animal models in order to clarify the regulatory mechanism of Notch signaling on MMP-13 through Runx2. Finally, we investigated whether the regulatory effect of Notch signaling on MMP-13 expression via Runx2 still exists under the stimulus of IL-1β.

## Materials and Methods

### Animals

14-day-old and 12-week-old male Sprague-Dawly (SD) rats were obtained from the Experimental Animal center of Sichuan University, China. Rats were randomly allocated to treatment groups/harvest time-points, fed with tap water and pellet food for easy access under standard temperature and humidity conditions, and killed by pulling their necks. In the *in vitro* studies, qRT-PCR, western and staining experiments were performed on n = 3/group/time-point. In the *in vivo* studies, staining experiments on lentivirus/Jagged-1/DAPT injected cartilage samples were performed on n = 6 rats/group. The experimental protocol was approved by the Animal Ethics Committee of the university (Sichuan University, Chengdu, China) and was carried out according to the Guidelines for the Care and Use of Laboratory Animals.

### Isolation of chondrocytes and cell culture

Chondrocytes were prepared from knee cartilage tissues of 14-day-old rats. Briefly, harvested cartilage tissues were cut into small pieces (<2 mm^3^) and were digested with 0.2% trypsin (Hyclone, USA) and collagenase (Sigma-Aldrich, USA). Then a cell suspension was yielded. Isolated chondrocytes were suspended in DMEM medium supplemented with 10% FBS (Hyclone, USA) and were counted in hemocytometer. The chondrocytes were seeded on a 90-mm petri dish at a density of 1.5 × 10^5^/dish, and passaged approximately at a 7-day interval. Chondrocytes at P3 stage, as mature chondrocytes, were taken for subsequent experiments. Characterization of the chondrocytes in primary cultures was established by immunostaining of Col II and toluidine blue staining (data not shown).

### Transfection of Runx2 interference lentivirus vector into chondrocytes and rat knee joint cartilage

The lentivirus vector with Runx2 shRNA (ID NM_053470) was constructed by Genechem Co.,Ltd (Shanghai, China). The oligonucleotide sequences were designed and synthesized as follows: Runx2-shRNA-F: 5′-CCGGCAGCACGCTATTAAATCCAAACTCGAGTTTGGATTTAATAGCGTGCTGTTTTTg-3′, Runx2-shRNA-R: 5′-AATTCAAAAACAGCACGCTATTAAATCCAAACTCGAGTTTGGATTTAATAGCGTGCtg-3′. The combined sequences of the EGFP gene and Runx2 shRNA were cloned into the *Ascl* and *Pmel* sites of the pGCSIL vector containing a CMV-driven GFP reporter (shRunx2). Scrambled shRNA unrelated to human gene sequences was used as a negative control (shControl).

*In vitro*, the chondrocytes were seeded before transfection at 5 × 10^4^ cells/well in 24-well plates and incubated in serum-free growth medium with polybrene (8 μg/ml). When cells reached 50% confluency, they were transfected with the shRunx2/shControl lentivirus at a multiplicity of infection of 100 (MOI = 100). Three days later, the transfection efficiency was measured by the percentage of GFP-positive cells/total cells, and the knocking-down efficiency was analyzed by qRT-PCR and western blot.

*In vivo*, 20 μl carrier solution with shRunx2/shControl lentivirus (10^8^tu/ml in the carrier solution) was slowly injected into the right articular cavity. 2 weeks later, rats were used for further injection treatment.

### Construction of Runx2 over-expression vectors

Runx2 over-expression vector construction as previously described^21^. Briefly, total RNA prepared from MC3T3-E1 cells was used to generate complementary DNAs for the Runx2 gene using PrimeScript™ Reverse Transcriptase reagent kit (2680 A, TaKaRa, Japan). cDNA was confirmed by DNA sequencing. After digestion with BglII and NotI (R6081 & R6431, Promega, USA), purification from agarose gel, PCR products was subcloned into pCMV-myc-C vector (635689, Clonteth, USA). The Runx2 plasmid DNA was transfected into cells (OE-Runx2) using Lipofectamine™ 2000 Transfection Reagent (11668027, ThermoFisher, USA). Untreated Cells were used as control (OE-Control).

### Intervention of Notch signaling by adding exogenous recombinant protein Jagged-1 and DAPT *in vitro* and *in vivo*

*In vitro*, untreated cells (normal), Runx2 shRNA transfected cells (shRunx2) and scrambled shRNA transfected cells (shControl) were respectively divided into three groups for each test, including the group treated with exogenous recombinant rat protein Jagged-1 (599-JG, R&D, USA), DAPT (2634, R&D, USA) and DMSO (3176, R&D, USA, as control). At first, the working density of Jagged-1 was confirmed by a gradient test. 6 hours after Jagged-1 treatment, chondrocytes were harvested to test Notch1 mRNA expression by qRT-PCR, showing 0.5 μg/ml the most effective density (*p* < 0.05) (Fig. S1). 100 μM DAPT solution was used according to manufacturer’s instructions. Solution of DMSO (1:10000 in PBS) was used as control^11,19^. Samples in each group were collected 6, 12, 24 and 48 hours after treatment respectively.

*In vivo*, drug injection dosages were followed as previously described^12^ and according to our *in vitro* data. 2 weeks after shRunx2 lentivirus injection, animals were started to receive weekly intra-articular injection with DAPT (0.3 ml, 10 µM), Jagged-1 (0.3 ml, 0.5 μg/ml) or DMSO (0.3 ml) as control. After 4 injections of DAPT/Jagged-1, animals were sacrificed 24 h after the last injection, then the knee joints were carefully dissected and fixed in 3.7% PFA at 4 °C for 48 hours. Thereafter samples were embedded in paraffin. 5 µm sections were cut on an HM360 microtome (Microm, Walldorf, Germany). Each one of every 5 consecutive sections were stained with hematoxylin (VWR, Rad- nor, PA, USA) and eosin (Sigma-Aldrich, Carlsbad, CA, USA) (H&E). Other sections were used for immunohistochemical staining.

### Viability of chondrocytes

The cell counting kit-8 (CCK-8, WST, Japan) was used for quantitatively evaluating the cell viability. Chondrocytes were seeded at 2 × 10^3^ cells/well into 96-well plates and were allowed to adhere to the plates overnight. Cells were then divided and treated with different stimuluses (Jagged-1, DAPT or DMSO as control) for 6, 12, 24 and 48 hours. 10 μl CCK-8 reagent was added to each well and incubated for another 4 hours. The absorbance was read (wavelength = 450 nm). Data of cell proliferation was assessed based on the average absorbance values of each group, according to the manufacturer’s protocol.

### EdU labeling of DNA replicated in chondrocytes

A Click-iT® EdU imaging kits (Invitrogen, USA) was used for measuring proliferation capacity of chondrocytes. Chondrocytes were seeded in a 6-well plate at 10^5^/well. After Jagged-1/DAPT treatment, 10 μM EdU was added to the medium. Cells were then fixed with 3.7% formaldehyde, and were incubated with Click-iT reaction cocktail in dark. The nuclei were counterstained with DAPI (C1005, Beyotime, China). The stained cells were observed using a fluorescence microscope (Olympus, Japan). Percentage of EdU-positive cells was calculated by number of red-fluorescent (Alexa 594-stained) cells/ number of DAPI-stained cells.

### RNA extraction and qRT-PCR

Total RNA was isolated using TRIZOL reagent (Invitrogen, USA), chloroform and isopropanol. Reverse transcription of RNA was performed by RevertAid^TM^First Strand cDNA Synthesis Kit (K1622, Thermo Fisher, USA). qRT-PCR with cDNA was performed using 2xPCR Master Mix (K0172, Thermo Fisher, USA). Gene expression relative to GAPDH was calculated using the 2^−ΔΔCT^ formula method. The sequences of primers are shown in Table 1.

**Table 1:** Primer sequences used in qRT-PCR analyses.

Gene	Forward	Reverse	Gene Number	Product length
Notch1	5′-ACTGCCCTCTGCCCTATACA-3′	5′-GACACGGGCTTTTCACACAC-3′	NM_001105721	189 bp
Hes 5	5′-AAGGCCGACATCCTGGAGAT-3′	5′-CGAGTAACCCTCGCTGTAGTC-3′	NM_024383	108 bp
Hes 1	5′-TCAACACGACACCGGACAAA-3′	5′-GGAATGCCGGGAGCTATCTT-3′	NM_024360	158 bp
Col X	5′-TGATCCTGGAGTGGGAGGAG-3′	5′-GGGATACCTGGTGGTCCAAT-3′	XM_001053056	150 bp
Sox 9	5′-CTCCTACTACAGCCACGCAG-3′	5′-GCTGTGTGTAGACGGGTTGT-3′	NM_080403	170 bp
MMP-13	5′-ACCCAGCCCTATCCCTTGAT-3′	5′-TCTCGGGATGGATGCTCGTA-3′	NM_133530	178 bp
Runx2	5′-TCCCAGTATGAGAGTAGGTGTCC-3′	5′-GGCTCAGATAAGAGGGGTAAGAC-3′	NM_001278483	164 bp
Col II	5′-GGCCAGGATGCCCGAAAATTA-3′	5′-GGCTGCAAAGTTTCCTCCAC-3′	NM_012929	267 bp
GAPDH	5′-TATGACTCTACCCACGGCAAGT-3′	5′-ATACTCAGCACCAGCATCACC-3′	NM_017008	138 bp

### Protein isolation and Western blot analyses

The total protein was collected using a protein extraction kit (Sigma-Aldrich, USA). The protein concentrations were determined using the BCA assay (Pierce, Thermo Fisher, USA). After denaturing at 95 °C for 5 mins, 5 mg aliquot of total protein of each sample was subjected to the 4–20% gradient SDS-PAGE, transferred to a PVDF membrane (Millipore, USA). The membrane was incubated in blocking solution with donkey serum (GTX30972, GeneTex, USA) for 1 hour, and was incubated overnight at 4 °C with the primary antibodies against Col X (ab58632, Abcam, USA), Col I (ab6308, Abcam, USA), Col II (ab116242, Abcam, USA), Sox 9 (PA5-23383, ThermoFisher, USA), Aggrecan (MA3-16888, ThermoFisher, USA), Notch1-ICD (07–1232, Millipore, USA), MMP-13 (MAB13426, Millipore, CA), Runx2 (sc-10758, Santa Cruz, USA) and β-actin (AA128, Beyotime, China). One of the following secondary antibodies was used: HRP-labeled goat anti-rabbit IgG (A0208, Beyotime, China) and HRP-labeled goat anti-mouse IgG (A0216, Beyotime, China). Proteins in the blots were visualized by an ECL plus kit (P0018, Beyotime, China). The integrated optical density of bands was quantified with the ImageJ software. Each sample was normalized to β-actin.

### Immunofluorescent staining (IFS) and Immunohistochemical staining (IHC)

For *in vitro* IFS, chondrocytes were fixed with 3.7% paraformaldehyde at first. Endogenous peroxidase activity was depleted with 3% hydrogen peroxide. then nonspecific antibody binding was blocked. The cells were incubated with a mouse monoclonal anti-MMP-13 (MAB13426, Millipore, CA) as primary antibody, and a Cy3-labeled goat Anti-Mouse IgG (A0521, Beyotime, China) as secondary antibody. Nuclei was counterstained with DAPI (C1005, Beyotime, China). The fluorescence stained cells were observed under fluorescence microscope (Olympus, Japan).

For *in vivo* IFS and IHC, after dewaxing in xylene, sections were rehydrated in an alcohol series (100, 100, 95, and 95%). Briefly, endogenous peroxidase activity was depleted with 3% hydrogen peroxide. then nonspecific antibody binding was blocked. The sections were respectively incubated with rabbit anti-GFP antibody (ab183735, Abcam, USA) and mouse monoclonal anti-MMP-13 (MAB13426, Millipore, CA) as the primary antibodies overnight at 4 °C. In immunofluorescent staining, sections were stained with a goat anti rabbit IgG H&L (FITC) secondary antibody (ab6717, Abcam, USA). DAPI (C1005, Beyotime, China) was used for nuclear counterstaining. For immunohistochemistry staining, the tissue was stained with HRP labeled goat anti-mouse IgG (H + L) (A0216, Beyotime, China) followed by visualization of HRP activity by a DAB-HRP color development kit (P0202, Beyotime, China).

### Statistical analyses

All data are presented as mean ± standard deviation. Statistical significance among the groups with a single variable was assessed using one-way ANOVA analysis with Tukey’s test. The other statistical significance among groups was assessed using two-way ANOVA analysis with multiple comparisons. Analyses were performed using the SPSS 17.0 statistical software program. All experiments were performed at least thrice in triplicate. A *p* value < 0.05 was considered to be the level of significance.

## Results

### Expression of Nothch1 was enhanced by Jagged-1 and inhibited by DAPT *in Vitro*

**Figure 1:**
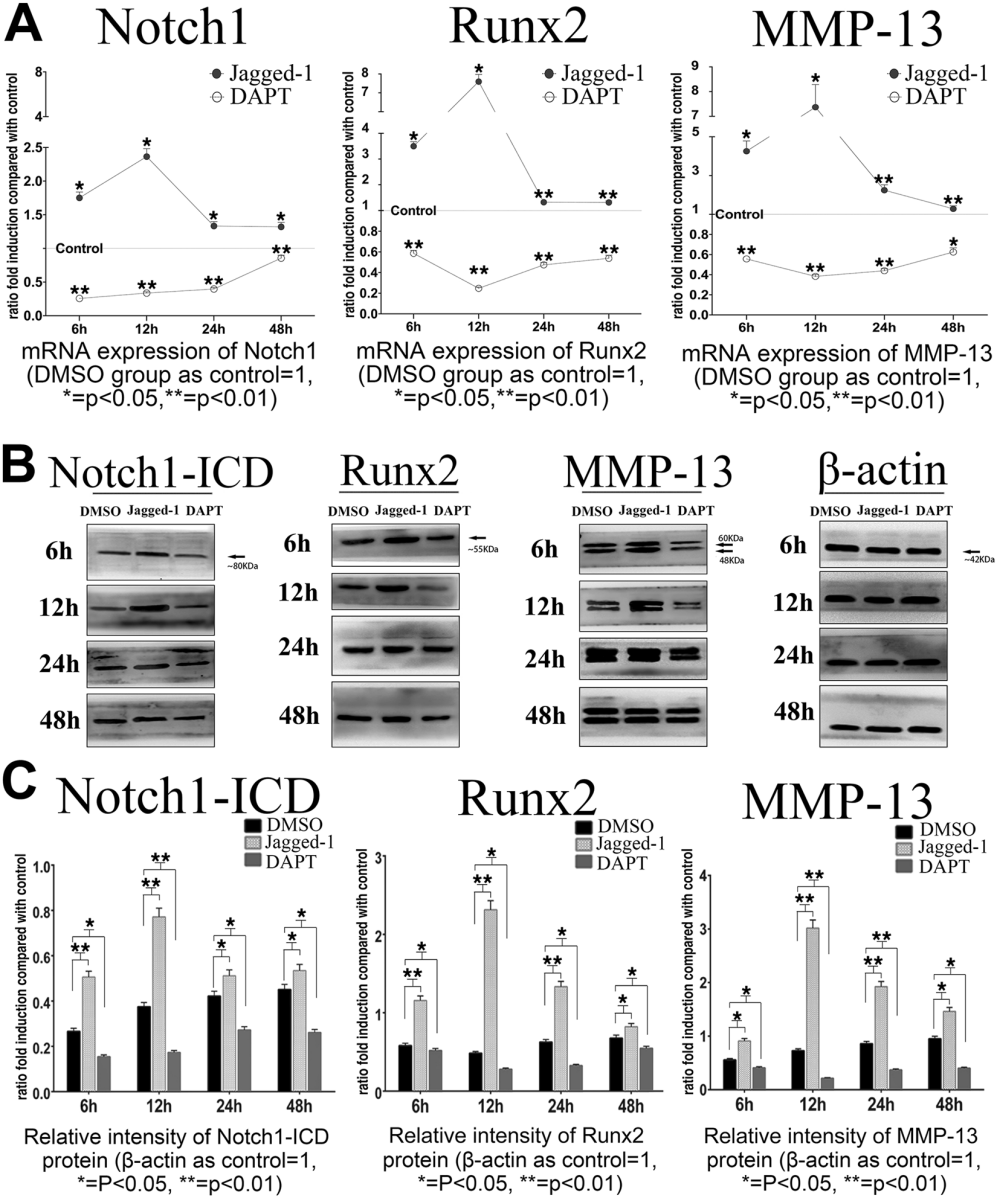
(**A**) mRNA expressions of Notch1, Runx2 and MMP-13 after Jagged-1 and DAPT treatments within 48 hours. (**B**,**C**) protein expressions of Notch1-ICD, Runx2 and MMP-13 within 48 hours after Jagged-1 and DAPT treatment. (N = 3). Full images of immunoblots in B were added in Supplemental Fig. 11.

To verify whether Notch signaling could be activated or suppressed in chondrocytes by Jagged-1/DAPT, expression of Notch1 was examined by qRT-PCR and western blot. After Jagged-1 treatment, Notch1 expression was increased at both mRNA (Fig. 1A) and protein levels (Fig. 1B,C). Expression of Notch1 reached up to 2.4-fold at mRNA level and 2-fold at protein level at 12 hours after Jagged-1 stimulation. On the other hand, Notch1 expressions were decreased both at mRNA level (by 74%) at 6 hours after DAPT treatment, and decreased at protein level (by 55%) at 12 hours after treatment (Fig. 1A–C). From 6 hours to 48 hours after Jagged-1 and DAPT treatment, Notch1 mRNA was gradually turned back to normal level, which was possibly attributed to a self-regulation of chondrocytes responding to external stimuli (Fig. 1A).

### Increased Notch signaling suppressed proliferation and stimulated differentiation in mature chondrocytes at early stage

**Figure 2:**
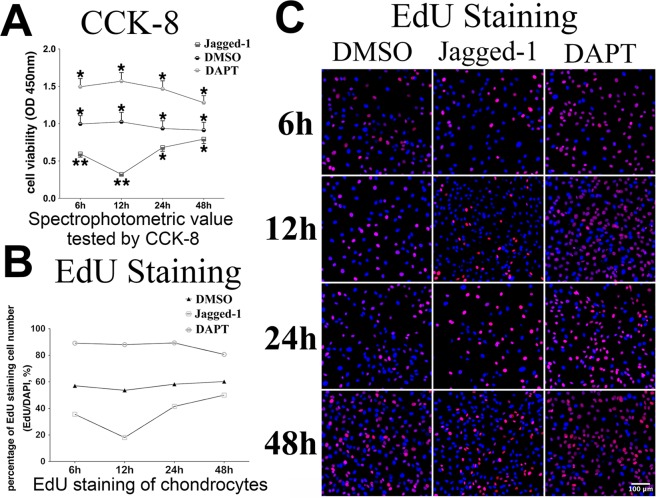
(**A**) CCK-8 analyses of chondrocytes within 48 hours after Jagged-1 and DAPT treatments. (**B**,**C**) EdU staining of chondrocytes within 48 hours after Jagged-1 and DAPT treatments. Red-fluorescent granules are EdU staining for nuclei of proliferative chondrocytes during the test period, and blue-fluorescent granules are DAPI staining for all cell nuclei under the identical observation area. (N = 3).

At the level of cytology, OA initiation is characterized by alterations of chondrocytes proliferation and differentiation. In order to investigate the influence of Notch signaling change on chondrocyte proliferation, we performed the CCK-8 test and EdU staining on chondrocytes *in vitro* after Jagged-1 or DAPT treatment. The CCK-8 test showed that the viability of chondrocytes decreased in Jagged-1 treated group and increased in DAPT treated group. Compared with control, prominent changes were observed at 12 hours after both treatments (decreased by 70% in Jagged-1 group and increased by 1.7-fold in DAPT group) (Fig. 2A). Consistently, EdU staining showed remarkably lower multiplication rate in Jagged-1 treated group (18–50%) and higher multiplication rate in DAPT group (80–89%) compared with control group (at about 60%) (Fig. 2B,C).

**Figure 3:**
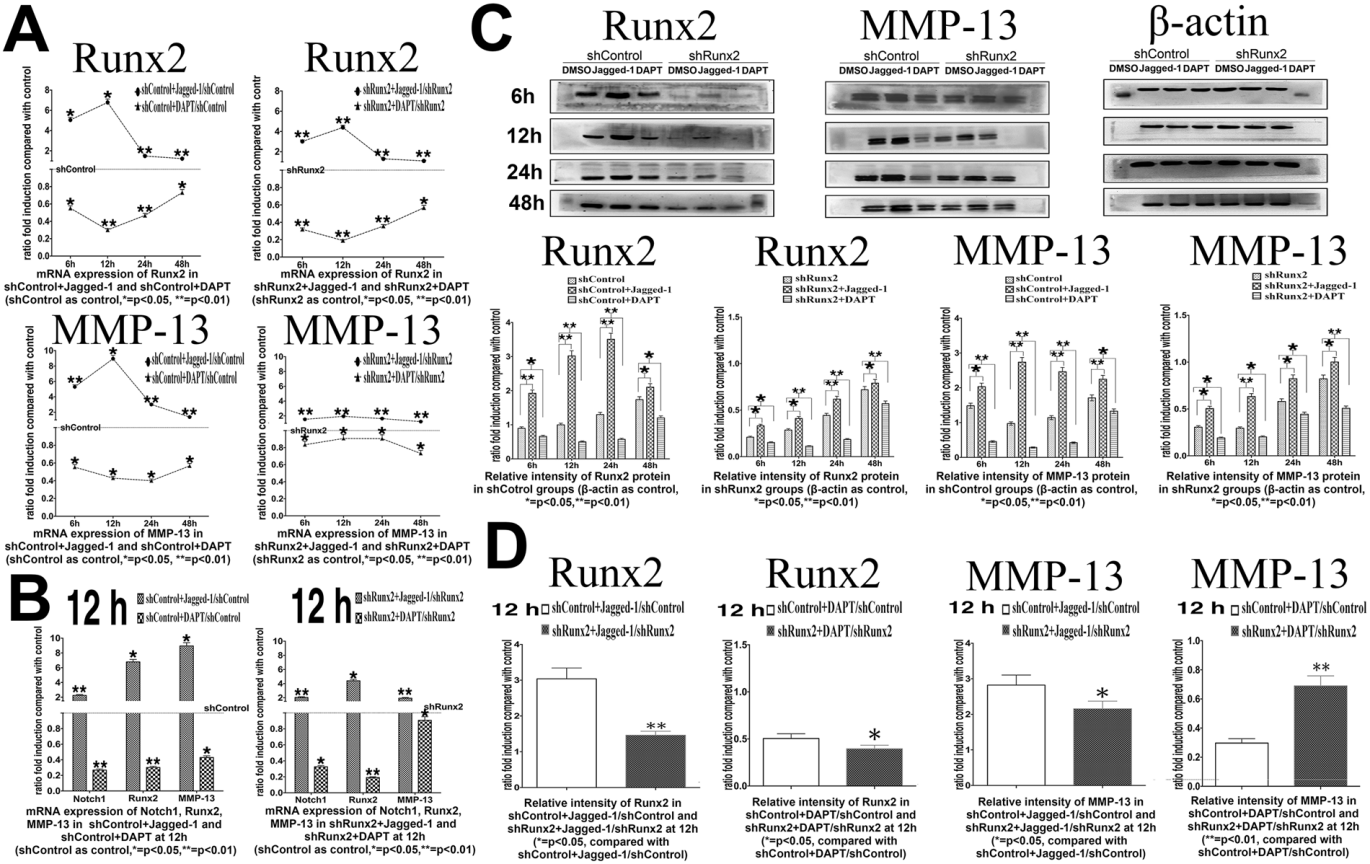
(**A**) mRNA expressions of Runx2 and MMP-13 in shControl group and shRunx2 group within 48 hours after Jagged-1 and DAPT treatment. Gene expressions in shControl group and shRunx2 group without Jagged-1/DAPT injection were set as baseline respectively. (**B**) mRNA expressions of Notch1, Runx2 and MMP-13 at 12 hours after Jagged-1 and DAPT treatments. (**C**) Protein expressions of Runx2 and MMP-13 in shControl group and shRunx2 group within 48 hours after Jagged-1 and DAPT treatment. Protein expression levels in shControl group and shRunx2 group without Jagged-1/DAPT injection were set as baseline respectively. (**D**) Change rates of Runx2 and MMP-13 protein expression levels in shControl group and shRunx2 group at 12 hours after Jagged-1 and DAPT treatments. The ratio of (shControl + Jagged-1/shControl) and (shControl + DAPT/shControl) were set as control groups respectively. (N = 3).

To investigate the effect of Notch signaling changes on chondrocyte differentiation, we analyzed hypertrophic and osteogenic differentiation markers of chondrocytes. Expression of Runx2, Col X and MMP-13, were significantly activated by Jagged-1 treatment (reached up to 7.6/6.5/7.3-fold at mRNA level and to 4.8/3.6/4.2-fold at protein level vs. control, *p* < 0.05), whereas DAPT treatment significantly reduced their expression (decreased down to 48%/48%/62% at mRNA level and 42%/58%/72% at protein level vs. control, *p* < 0.01) (Fig. 3, S2A,B). On the contrary, expression of Sox9 (a negative regulative marker for chondrocytes hypertrophy) and Col II were significantly decreased in Jagged-1 treated group (decreased down to 72%/63% at mRNA level and to 66%/55% at protein level vs. control) and were increased in DAPT treated group (increased up to 1.6-/1.7-fold at mRNA level and 1.6-/2.5-fold at protein level vs. control). (Fig. S2A,B). These findings indicated that activation of Notch signaling inhibited proliferation and promoted hypertrophic differentiation in mature chondrocytes at early stage.

### Notch signaling partly regulated the expression of MMP-13 via transcriptional factor Runx2 *in vitro*

Our results revealed that activation of Notch signaling significantly increased the expression of Runx2 and MMP-13 at both mRNA and protein levels. In the meanwhile, inhibition of Notch signaling lead to notably decreased Runx2 and MMP-13 expressions (Fig. 1A–C) . Next, we suppressed Runx2 expression by RNA interference to identify whether this transcription factor was involved in the regulation of Notch signaling on MMP-13. We found there were about 86% decrease at mRNA level and 82% decrease at protein level of Runx2 in chondrocytes after shRunx2 lentivirus transfection (shRunx2 vs. shControl, p < 0.01), thereby confirming the effectiveness of Runx2 transcription interference (Figs S3A, 4A). Then, cells were treated with Jagged-1/DAPT. During the 48 h observation period after treatment, Runx2 interference did not significantly affect the function of Jagged-1 and DAPT on regulating Notch1 expressions (*p* > 0.05) (Fig. S5). At the same time, Notch signaling change by Jagged-1 or DAPT treatment could still regulate the expression of residual Runx2 genes (shControl + jagged-1/shControl vs. shRunx2 + jagged-1/shRunx2, shControl + DAPT/shControl vs. shRunx2 + DAPT/shRunx2) (Fig. 3B,D).

**Figure 4:**
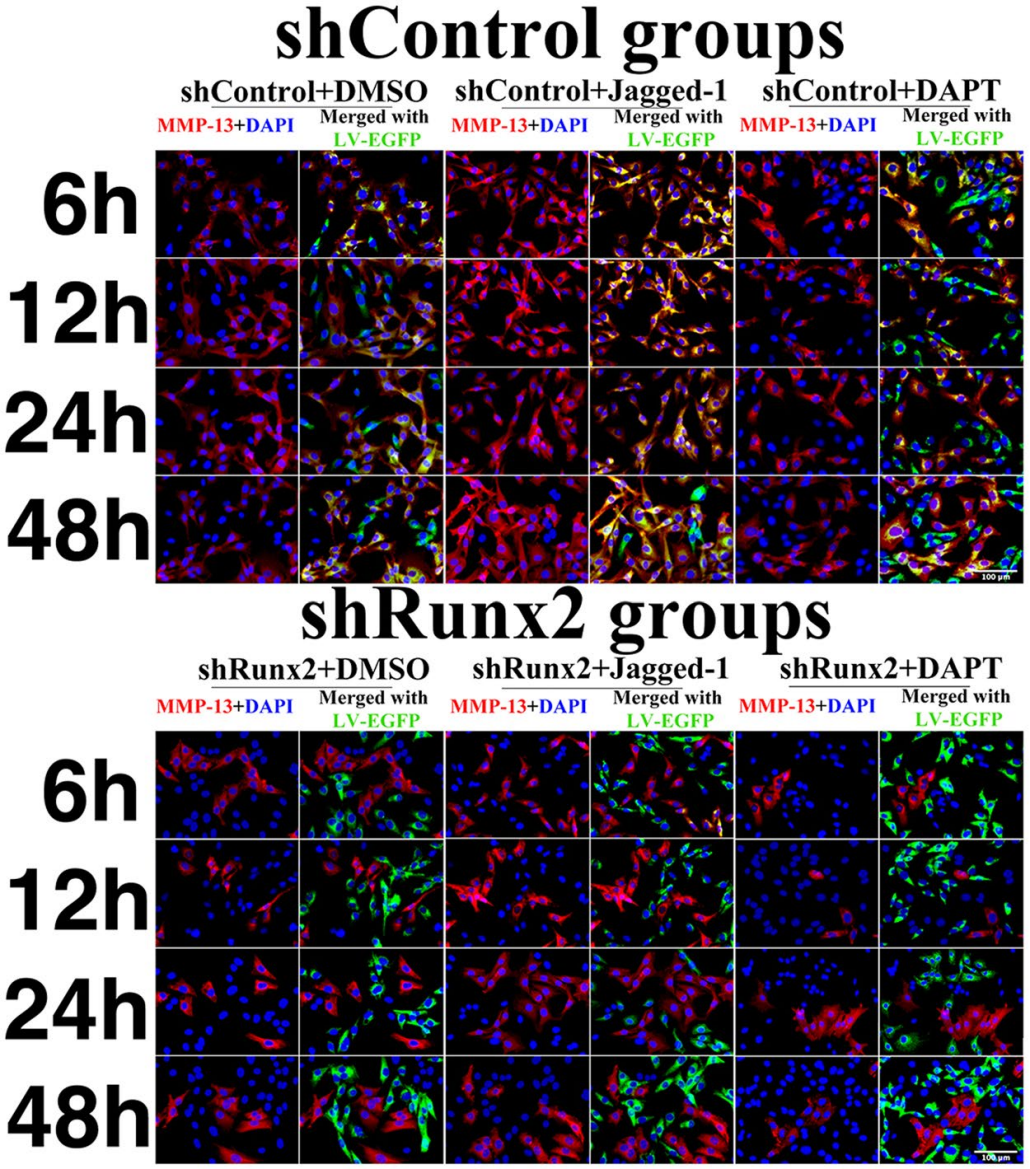
MMP-13 immunofluorescent staining in chondrocytes within 48 hours after Jagged-1 and DAPT treatments. Red fluorescence = MMP-13, green fluorescence = shControl or shRunx2 vector GFP.

In shControl group, MMP-13 expressions at both mRNA and protein levels were elevated by Jagged-1 (shControl + Jagged-1/shControl >1) (Fig. 3A). However, when Runx2 gene was knocked-down, the increase rate of MMP-13 mRNA and protein expressions were much less after Jagged-1 treatment. (*p* < 0.01 in mRNA, p < 0.05 in protein, shRunx2 + jagged-1/shRunx2 vs. shControl + jagged-1/shControl). In contrast, the decrease rate of MMP13 expression treated by DAPT was also attenuated after Runx2 knocking-down. Especially when MMP13 expression was most dramatically changed at 12 hours after Jagged-1/DAPT treatment in the control group, the knocking-down of Runx2 remarkably abated the regulation effect of Jagged-1/DAPT treatment on MMP13 (*p* < 0.05 in mRNA, p < 0.01 in protein, shRunx2 + DAPT/shRunx2 vs. shControl + DAPT/shControl) (Fig. 3A–D). To directly observe the expression of MMP-13 when Runx2 was knoked-down, we performed immunofluorescent staining. In the control group, MMP-13 expression was found increased by Jagged-1 treatment and was decreased by DAPT treatment. In the shRunx2 group, we found the extent of MMP13 expression changes was attenuated, which was consistent with our qPCR and Western blot quantification analyses (Fig. 4). However, although Runx2 protein expression was increased by 2 folds approximately in overexpression-Runx2 groups, we found there wasn’t significant changes of the ‘regulatory effect’ of Notch signaling activation/inhibition on MMP13 expressions after Runx2 over-expression. Under our experimental condition, we supposed that endogenous Runx2 in chondrocytes was essential and enough to maintain Notch signaling transduction to regulate MMP13 expression (Fig. S8). These findings demonstrated that interference of Runx2 expression caused a partial failure of regulation on MMP-13 by Notch signaling changes.

### Regulatory effects of Jagged-1 and DAPT on MMP-13 expression were abated by knocking down Runx2 in rat articular cartilage

**Figure 5:**
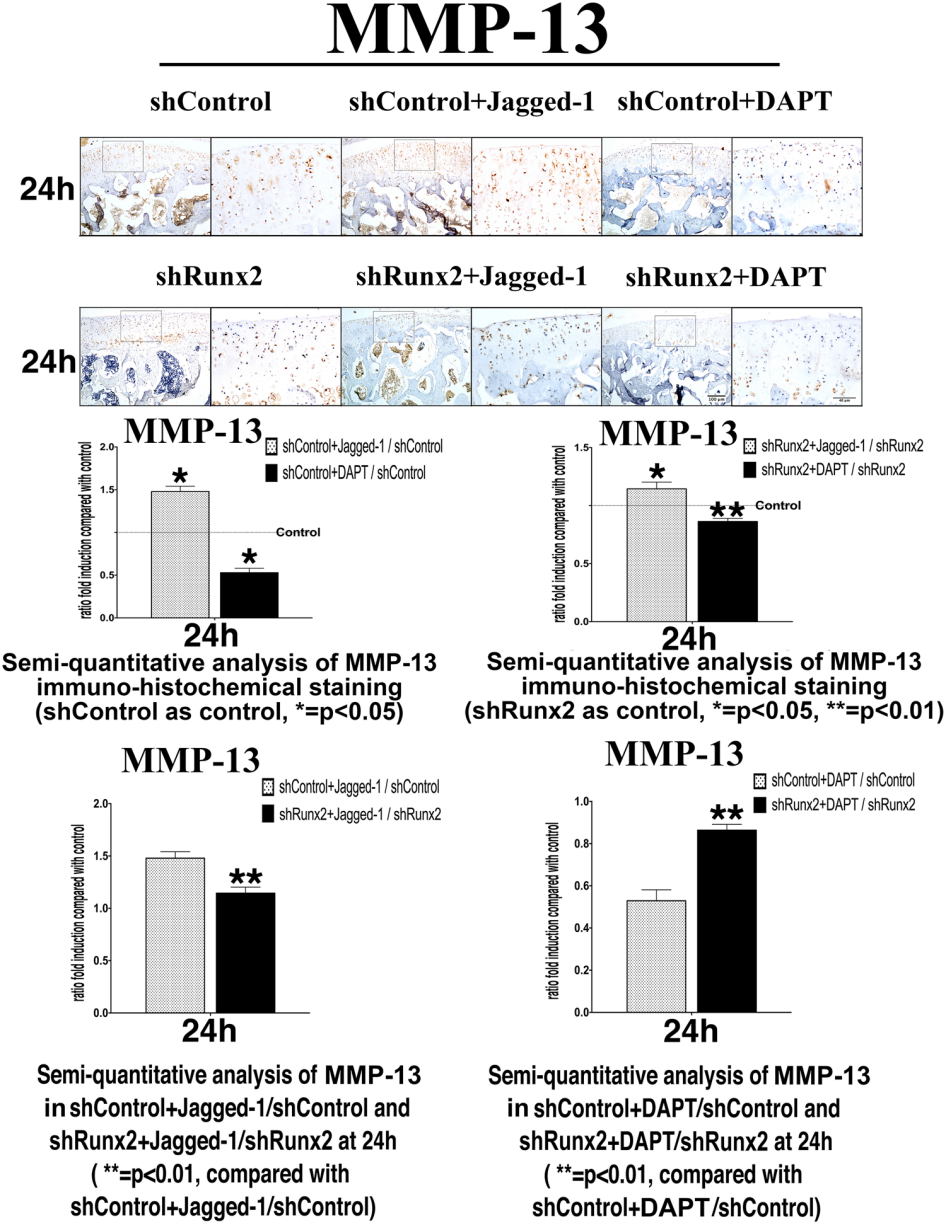
Immunohistochemical staining of rat knee articular cartilages and semi quantifications on the change rates of MMP13 positive cells at 24 h after Jagged-1 and DAPT treatments. The positive cells were showed as cell number ratio (shControl + (Jagged-1 or DAPT)/shControl, shRunx2 + (Jagged-1 or DAPT)/shRunx2), protein expression levels in shControl group and shRunx2 group without Jagged-1/DAPT injection were set as baseline respectively. Compared with shControl + Jagged-1/shControl, the ratio of MMP-13 protein expression levels was decreased in shRunx2 + Jagged-1/shRunx2. On the contrary, after DAPT treated, the ratio of MMP-13 protein levels expression was increased in shRunx2 + DAPT/shRunx2 in comparation with shControl + DAPT/shControl. (N = 6).

To further verify our *in vitro* findings, we performed Jagged-1 and DAPT intra-articular injection in the knee joint of SD rats, and suppressed the Runx2 expression by local injection of shRunx2 lentivirus. After lentivirus injection, Runx2 was successfully knocked-down by approximately 75% in rat knee articular cartilage (Fig. S3B). Similar changes of MMP13 expressions to the *in vitro* quantification analyses were observed, including significantly increased MMP13 expression activated by Jagged-1 and decreased MMP13 expression restrained by DAPT in the surface of articular cartilage. When Runx2 was interfered, regulation on MMP13 expression by Notch signaling change through Jagged-1/ DAPT became subdued (Fig. 5).

Besides, by IHC staining, we found that Col II and Sox 9 expressions were reduced by Jagged-1 treatment and were increased by DAPT treatment in both shControl and shRunx2 groups. Moreover, as the major structural proteoglycan was found in the extracellular matrix of cartilage, aggrecan was increased or decreased after Jagged-1/DAPT treatment respectively (Figs S9, S10).

### Notch signaling is involved in MMP-13 expression mediated by IL-1β

In above experiments, we proved that enhanced or inhibited Notch signaling in normal mature chondrocytes led to an increase or a decrease of MMP-13 expression, the vital collagenase during initiation and progression of OA. This encouraged us to presume that Notch signaling could also regulate MMP-13 expression within chondrocytes under an inflammatory environment. To test this hypothesis, we treated mature chondrocytes with both IL-1β (501-RL, R&D, USA) and DAPT. These cells were further divided to shControl or shRunx2 group. Notch1, Runx2, and MMP-13 expression were examined within 48 h.

**Figure 6:**
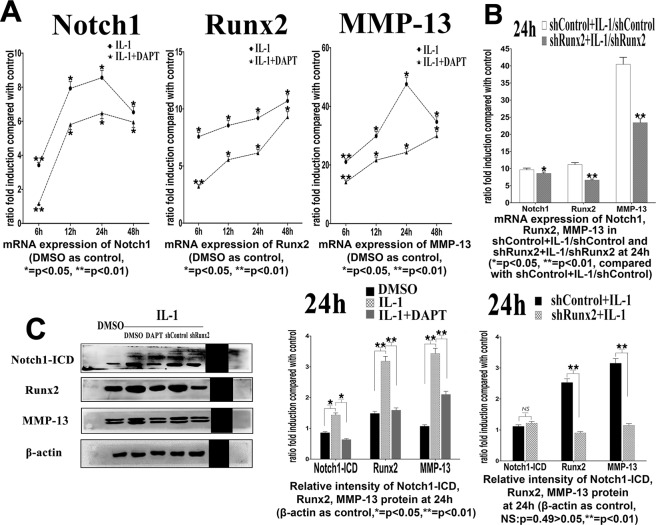
(**A**) mRNA expression of Notch1, Runx2 and MMP-13 under IL-1β stimulation within 48 hours after Jagged-1 and DAPT treatments. values were showed as ratio fold to DMSO control group. (**B**) mRNA expressions of Notch1, Runx2 and MMP-13 under IL-1β stimulation at 24 hours after Jagge-1 and DAPT treatments. (**C**) Protein expressions of Notch1-ICD, Runx2 and MMP-13 under IL-1β stimulation at 24 hours after Jagged-1 and DAPT treatments. (N = 3).

Compared with the control group, mRNA and protein expressions of Notch1/Notch1-ICD, Runx2 and MMP-13 were significantly up-regulated in chondrocytes treated with IL-1β (Fig. 6A). The MMP-13 mRNA expression was elevated up to approximately 48-fold (24 hours after treatment) and the protein expression was elevated up to about 3.2-fold (48 hours after treatment) (*p* < 0.05). Such increased expression of MMP-13 was down-regulated by DAPT to about 50% at mRNA level and 60% at protein level respectively (*p* < 0.05) (Fig. 6A,C). These results illustrated that suppression of Notch signaling decreased expression of MMP-13 induced by IL-1β.

When Runx2 was knocked-down, MMP13 expression under IL-1β circumstance could be significantly reduced (Fig. 6B,D, Figs S6, S7). Moreover, the ‘regulatory sensitivity’ of IL-1β on activating MMP-13 expression in chondrocytes was also notably suppressed by knocking-down Runx2. At 24 hours after IL-1 stimulation, the increase rate of MMP-13 expression was decreased by 53% at mRNA level and by 22% at protein level (*p* < 0.05, shRunx2 + IL-1/shRunx2 vs. shControl + IL-1/shControl) (Fig. 6B,D). Our results illustrated that the regulation of MMP-13 by Notch signaling activation induced by IL-1β was also partly mediated by a proper function of Runx2.

## Discussion

In normal articular cartilage surface, the immuno-activity of Notch receptors was undetectable. While in OA cartilage, Notch signaling is activated, and a much more abundant expression in the damaged areas was detected^22^. At the beginning of OA, the earliest degradation changes also occurred at the cartilage surface^23,24^. Accordingly, we speculated that the onset of OA is possibly associated with changes of Notch signaling pathway in chondrocytes, eventually resulting in an imbalance between production and degradation of ECM. To observe such early changes in OA, several time points within 48 h were chosen for observation after exogenous intervention of Notch signaling in mature chondrocytes.

### Early inhibition of proliferation and increase of hypertrophic differentiation in mature chondrocyte by activation of Notch signaling

In early OA, the normally quiescent chondrocytes undergo phenotypic shift, characterized by a transient proliferative response, a differentiation expressing hypertrophy-like changes and increased synthesis of cartilage-degrading enzymes, such as MMP-13^25,26^. But how Notch signaling are involved in this phenotypic change of chondrocytes remains controversial^16,27–30^. Some studies showed that Notch signaling is associated with significantly increased cell proliferation^16,28–30^. These results could be an explanation for the chondrocyte cloning found primarily in the upper cartilage zone, that is, in the same location as the Notch-1 ligand positive cells^31^. Another theory is that surface damage to the ECM might contribute to the increased proliferation^32^. Such response together with increased synthesis of ECM is considered as an early attempt to repair in early OA. In this study, we found that enhanced Notch signaling resulted in a decrease of chondrocytes proliferation. Such inhibitory effect could disrupt the self-regeneration of chondrocytes. On the other hand, our data showed hypertrophic differentiation markers, Col X, MMP-13 and Runx2, were significantly increased by activating Notch signaling, demonstrating that articular chondrocytes might lose their differentiated phenotype and obtain a behavior with similarities to terminal differentiating chondrocytes. Such terminal differentiation is defined as hypertrophy and apoptosis^33^. This process leads to degradation of cell surroundings, as can be seen in OA cartilages and OA models. Evidences above suggested that early suppression of proliferation and promotion of differentiation by activating Notch signaling could aggravate early OA. It is worth mentioning that when and how Notch signaling was activated may be the pivotal determinations to diverse outcomes^34,35^. Our results came from a transient alteration of Notch signaling in normal and mature chondrocytes, and detections were completed in an initial and short term. Thus, the controversial results between our study and previous studies were possibly due to different experimental conditions^16,28–30^.

### Activity of Notch signaling regulated expression of MMP-13 via the key factor Runx2

The first sign of prominent degeneration in OA is the onset of fibrillation at the articular cartilage surface as collagen fibrils had been damaged. Progressive denaturation of Col II was found in early OA, and a net loss of this molecule is accompanied with the cartilage damage^36^. During the degeneration process MMP-13 is known as the factor that preferentially cleaves Col II^37^. Our findings showed that when MMP-13 expression was up-regulated by Jagged-1 within 48 h, Col II expression was decreased and aggrecan was increased, which suggested that activity of Notch signaling was involved in the degradation of ECM during early OA by stimulating MMP-13 expression.

Indeed, the regulation of MMP-13 by Notch signaling is highly complex. Generally, sustained overexpression of Notch signaling increased abundance of MMP-13. Conversely, MMP-13 abundance decreased in conditionally overexpression of Notch signaling, which could not repair the injury of induced OA but exacerbated disease progression instead^34^. These evidences hinted that the role of Notch signaling in regulating MMP-13 expression depended on its dosage, as well as the duration and the context of the signaling activation. Interestingly, there is a putative CSL (Notch co-activator) binding site located in mouse and rats MMP-13 promoter sequence^38^. However, MMP-2 promoter area also has this binding site while MMP-2 was not regulated by Notch, suggesting that the regulation on MMP-13 by Notch signaling may achieved through a more circuitous mechanism^11^.

Runx2 is known as the direct upstream transcriptional regulator of MMP-13, and is regulated by Notch signaling in chondrocytes as well. In OA cartilage, Runx2 contributes to increase expression of MMP-13^9^. In Runx2^+/−^mice, OA development was resisted with decreased MMP-13 expression^10^. In addition, overexpression of Notch signaling in chondrogenic cell line significantly induced expression of Runx2^20^. In accordance with studies above, our data showed that transcriptional inhibition of Runx2 resulted in an attenuation of ‘regulation sensitivity’ of Notch signaling on MMP-13 expression. Our data, with other previous reports, suggested that early regulation of MMP-13 by Notch signaling could be partly mediated by Runx2 in mature chondrocytes.

DAPT is a small compound of Notch signaling inhibitor, which increases expression of Col II in the articular chondrocytes^39^. In a mouse surgical knee joints OA model, intra-articular injection of DAPT decreased expression of MMP-13, and the cartilage degradation was suppressed^19^. In our study, expression of MMP-13 induced by IL-1β was significantly suppressed by DAPT, highlighting that DAPT is a potential protector of Col II from degradation and might offer an optional therapy of clinical OA.

In conclusion, our findings demonstrated that activation of Notch signaling was involved in initial and development of early OA damage by suppressing proliferation and increasing hypertrophic differentiation in mature articular chondrocytes. Moreover, our data suggested that the expression of MMP-13 might be partly regulated by Notch signaling though the activation of Runx2 in chondrocytes under both normal and IL-1β circumstances. However, the animal model we used in this study was rat with intact normal cartilage, while animals with OA cartilage were not used in the current studies. Further work using OA animal models is therefore needed to better understand how Runx2 is involved in the Notch signaling transmission, and how these effects function in the cartilage degradation during AO progression.

## Supplementary information

Supplemental Figures
