# Supplementary material for: Notch Signaling Regulates MMP-13 Expression via Runx2 in Chondrocytes
Source: Sci Rep. 2019 Oct 30;9:15596. doi: 10.1038/s41598-019-52125-5 (PMC6821756; doi:10.1038/s41598-019-52125-5)
Supplement: Supplementary file 1 — Supplemental Figures [file 41598_2019_52125_MOESM1_ESM.pdf]

Notch Signaling Regulates MMP-13 Expression via Runx2 in Chondrocytes

Di Xiao[1,2]*, Ruiye Bi[1]*, Xianwen Liu[3], Jie Mei[1], Nan Jiang[1], Songsong Zhu[1#]

1. State Key Laboratory of Oral Diseases & National Clinical Research Center for Oral Diseases & Department of Orthognathic and TMJ Surgery, West China Hospital of Stomatology, Sichuan University, Chengdu, China.
2. Department of Stomatology, The First Affiliated Hospital of Dalian Medical University, Dalian, Liaoning, China.
3. Department of Oral and Maxillofacial Surgery, Guangdong Provincial Hospital of Stomatology, Southern Medical University, Guangzhou, China.

*These authors contribute equally to this work

#Corresponding Author: Songsong Zhu, DMD/PhD

No.14, 3rd section of Ren Min Nan Lu Road, Chengdu, China, 610000

Phone: (+86)02885502334

Fax: (+86)02885503530

Email: ZSS_1977@163.com

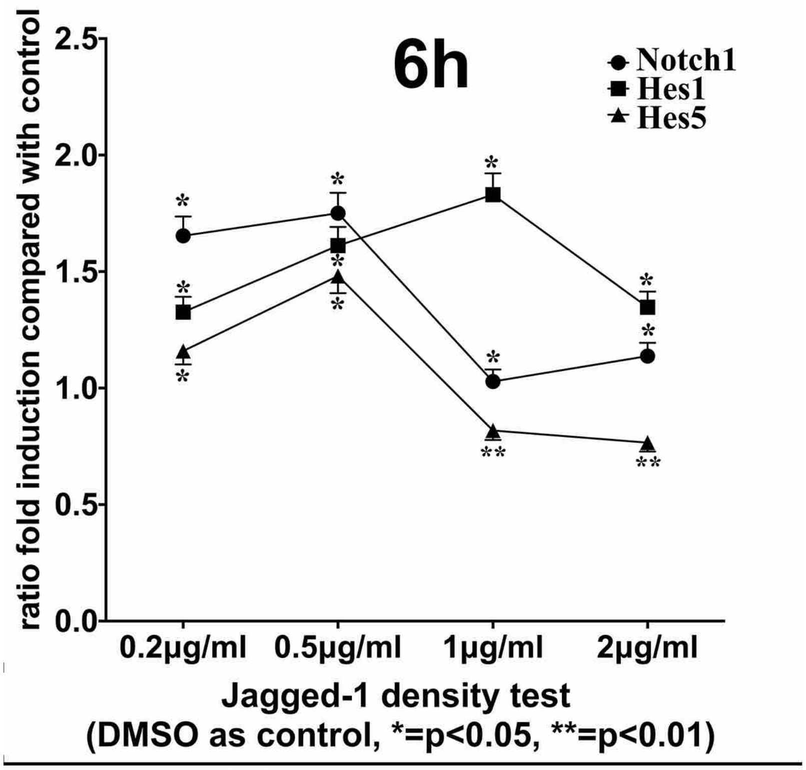

Supplemental Figure 1:
qRT-PCR results of Notch1 mRNA expression for the density test of Jagged-1 treatment. Results showed that a higher expression of Notch1 was observed at 6h after being treated with 0.5μg/ml Jagged-1 in chondrocytes.

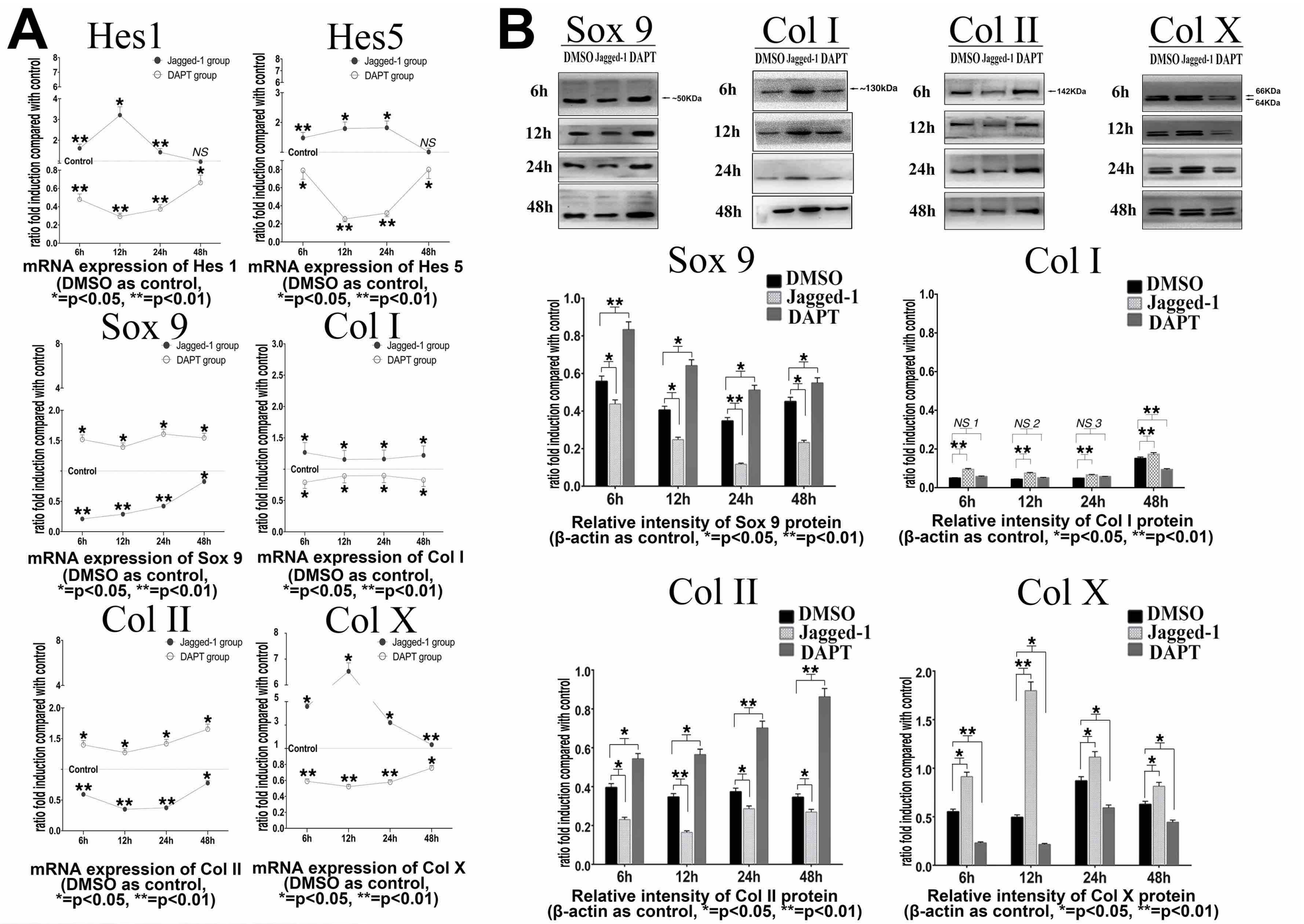

Supplemetal Figure 2:

A-B: mRNA expression of Hes1, Hes5, and mRNA and protein expression of Sox9, Col I, Col II, Col X within 48 hours after Jagged-1 and DAPT treatment. (In part A, NS in chart Hes1: p=0.7277>0.05; NS in chart Hes5: p=0.9417>0.05. In part B, in histogram Col I, NS1: p=0.0590>0.05; NS2: p=0.0760>0.05; NS3: p=0.0810>0.05.) Full images of immunoblots in B were added in Fig S11.

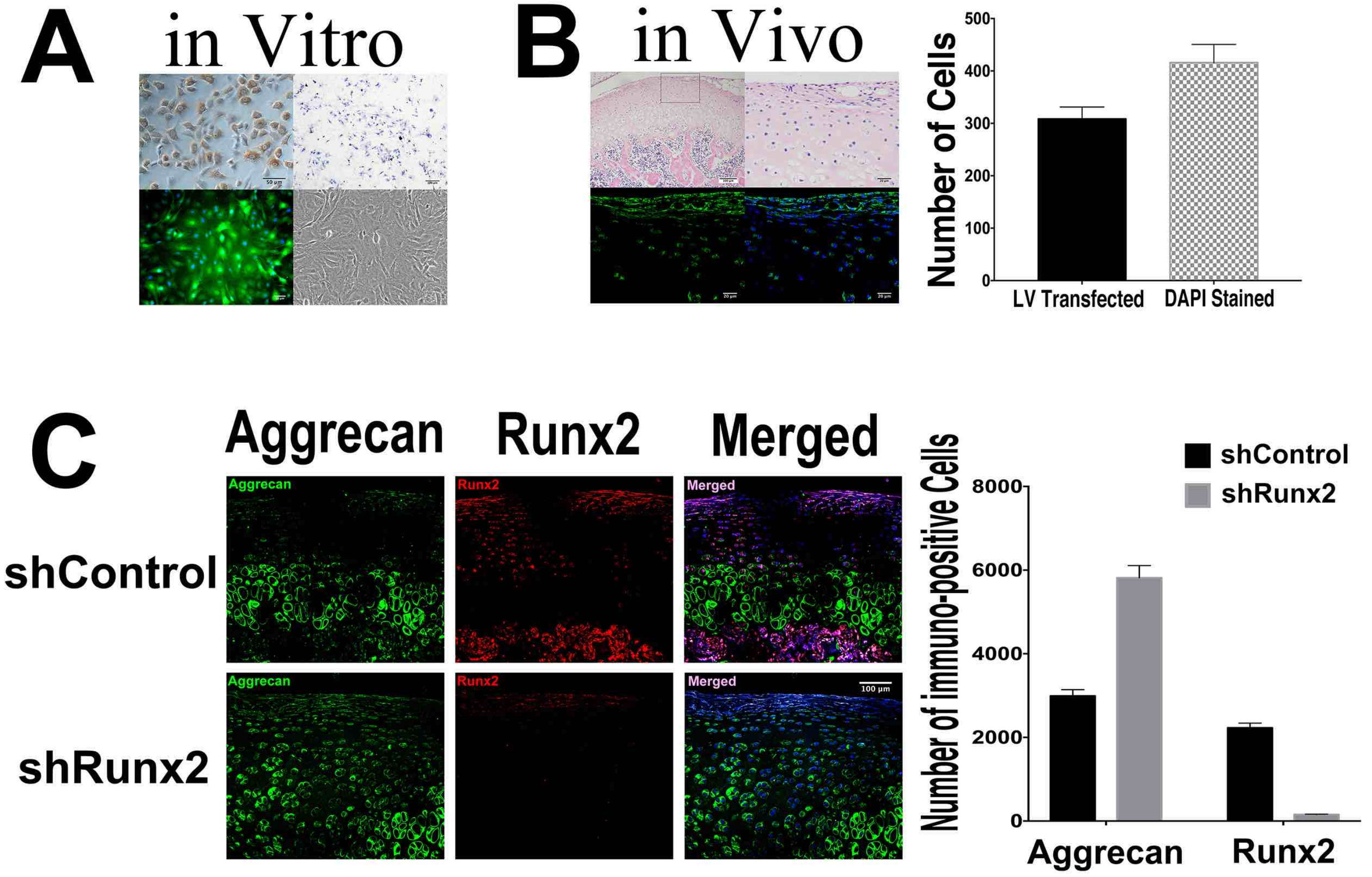

Supplemental Figure 3:

A: In Vitro, immunostaining of Col II and toluidine blue staining for confirming the primary chondrocytes of rat knee cartilage, and observation of lentivirus transfected chondrocytes under both fluorescence light and white light (green=vector GFP, blue=Dapi). B: In Vivo, H&E staining and GFP immunofluorescent staining of rat articular cartilage after shRunx2 lentivirus intra-articular injection. C: In Vivo, immunofluorescent staining of aggrecan and Runx2 in both shControl and shRunx2 groups.(green=aggrecan, red=Runx2)

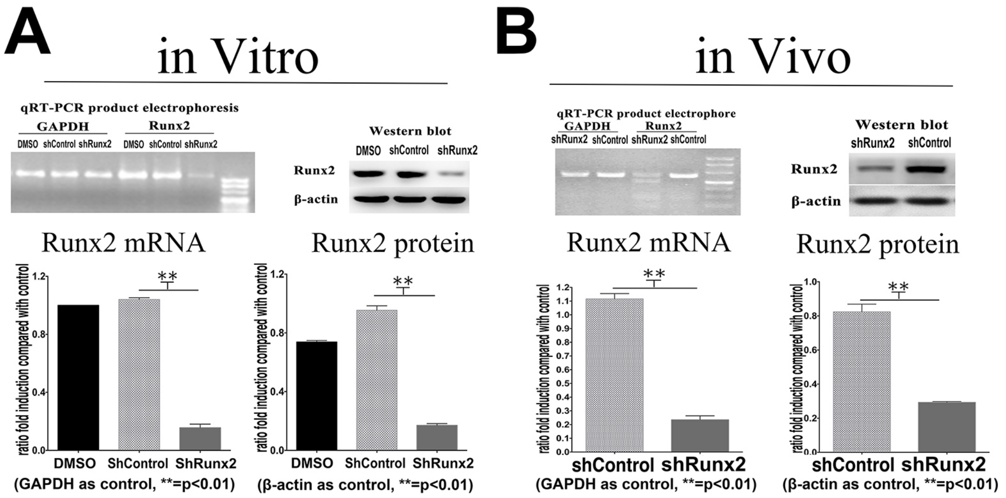

Supplemental Figure 4:

A: mRNA and protein expressions of Runx2 in chondrocytes in vitro 72 hours after shRunx2 transfection. B: mRNA and protein expressions of Runx2 in rat knee articular cartilage in vivo after shRunx2 knocking-down injections. Full images of immunoblots in A and B were added in Fig S11.

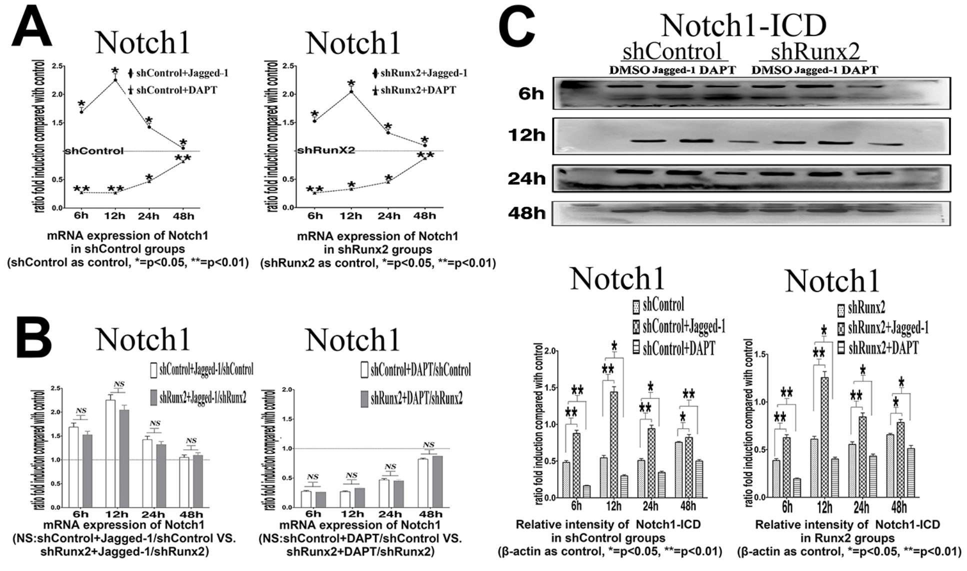

Supplemental Figure 5:
A: Ratio changes of Notch1 mRNA expression within 48 hours after Jagged-1 and DAPT treatments. B: Comparison of ratio changes of Notch1 mRNA expression between shControl groups and shRunx2 groups after Jagged-1 and DAPT treatment. C: Protein expression level changes of Notch1-ICD after Jagged-1 and DAPT treatments in both shControl groups and shRunx2 groups.(In part B, in histogram Notch 1 groups treated with Jagged-1, NS: p=0.140 >0.05; in histogram Notch 1 groups treated with DAPT, NS: p=0.410>0.05.)

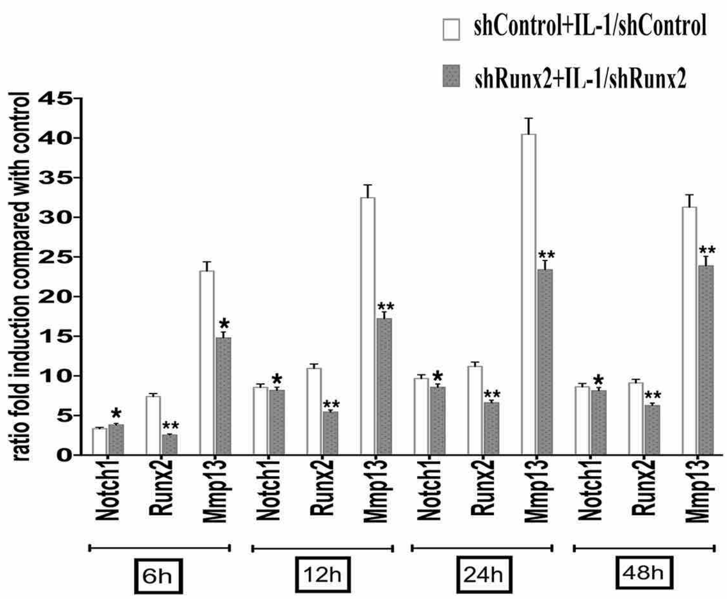

mRNA expression of Notch1, Runx2, MMP-13 in shControl+IL-1β/shControl and shRunx2+IL-1β/shRunx2 (*=p<0.05, **=p<0.01, compared with shControl+IL-1β/shControl)

Supplemental Figure 6:
Comparison of ratio changes of Notch1, Runx2 and MMP-13 mRNA expressions between shControl groups and shRunx2 groups under IL-1β stimulation.

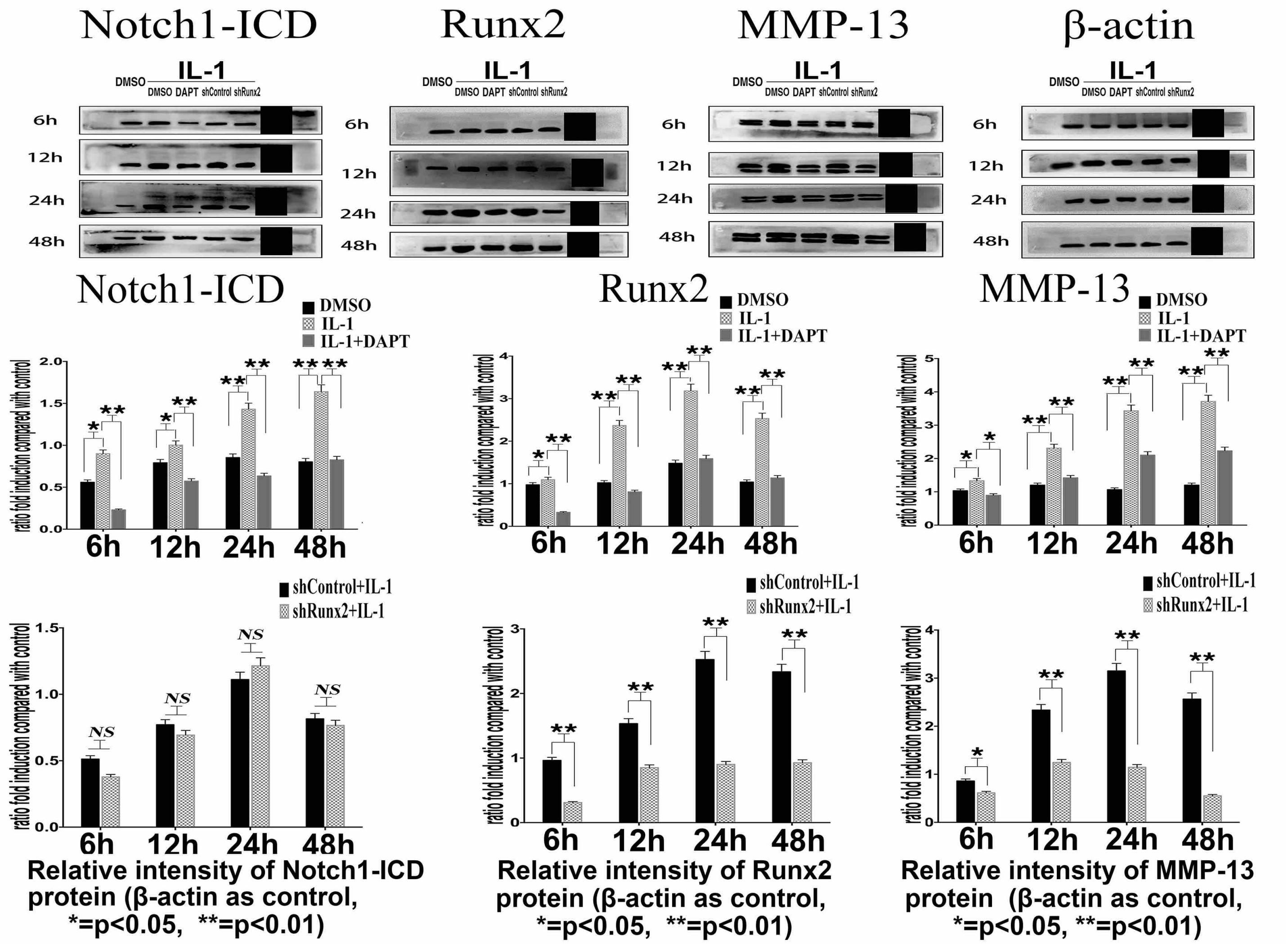

Supplemental Figure 7:
Protein expression changes of Notch1-ICD, Runx2 and MMP-13 in both shControl and shRunx2 groups within 48 hours after DAPT treatments under IL-1β stimulation. (In histogram Notch of shControl+IL-1 and shRunx2+IL-1 groups, NS: p=0.490>0.05.)

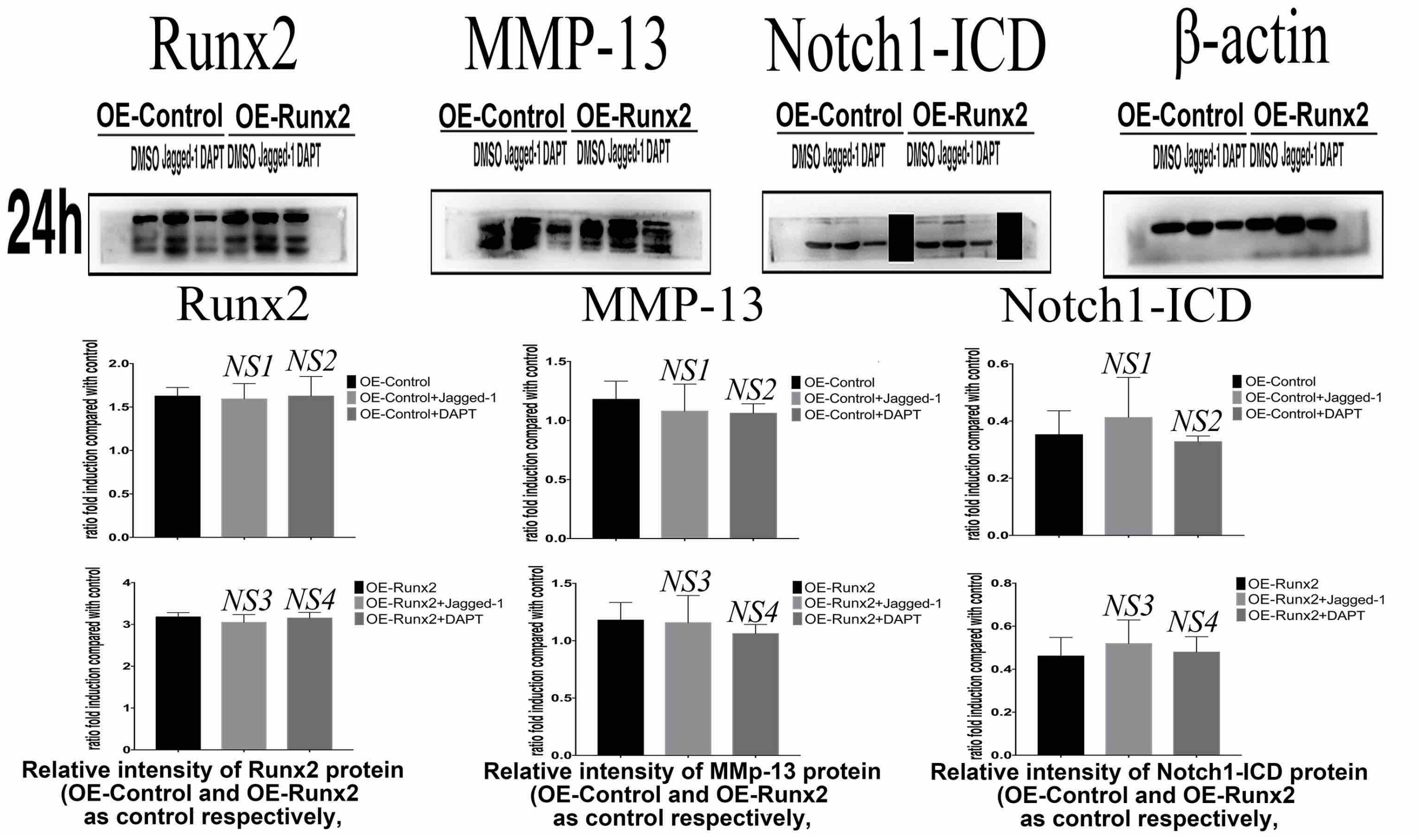

Supplemental Figure 8:
Protein expression of Runx2, MMP-13 and Notch1-ICD in OverExpression-Control groups and OverExpression-Runx2 groups in 24 hours after Jagged-1 and DAPT treatments. Protein expression levels in OE-Control groups and OE-Runx2 groups without Jagged-1/DAPT treatment were set as baseline respectively. (In histogram Runx2, NS1:p=0.6557>0.05; NS2:p=0.2940>0.05; NS3:p=0.0844>0.05; NS4:p=0.6253>0.05. In histogram MMP-13, NS1:p=0.2927>0.05; NS2:p=0.0586>0.05; NS3:p=0.2326>0.05; NS4: p=0.6345>0.05. In histogram Notch1-ICD, NS1:p=0.2889>0.05; NS2:p=0.4020 >0.05; NS3:p=0.1653>0.05; NS4:p=0.3689>0.05.)

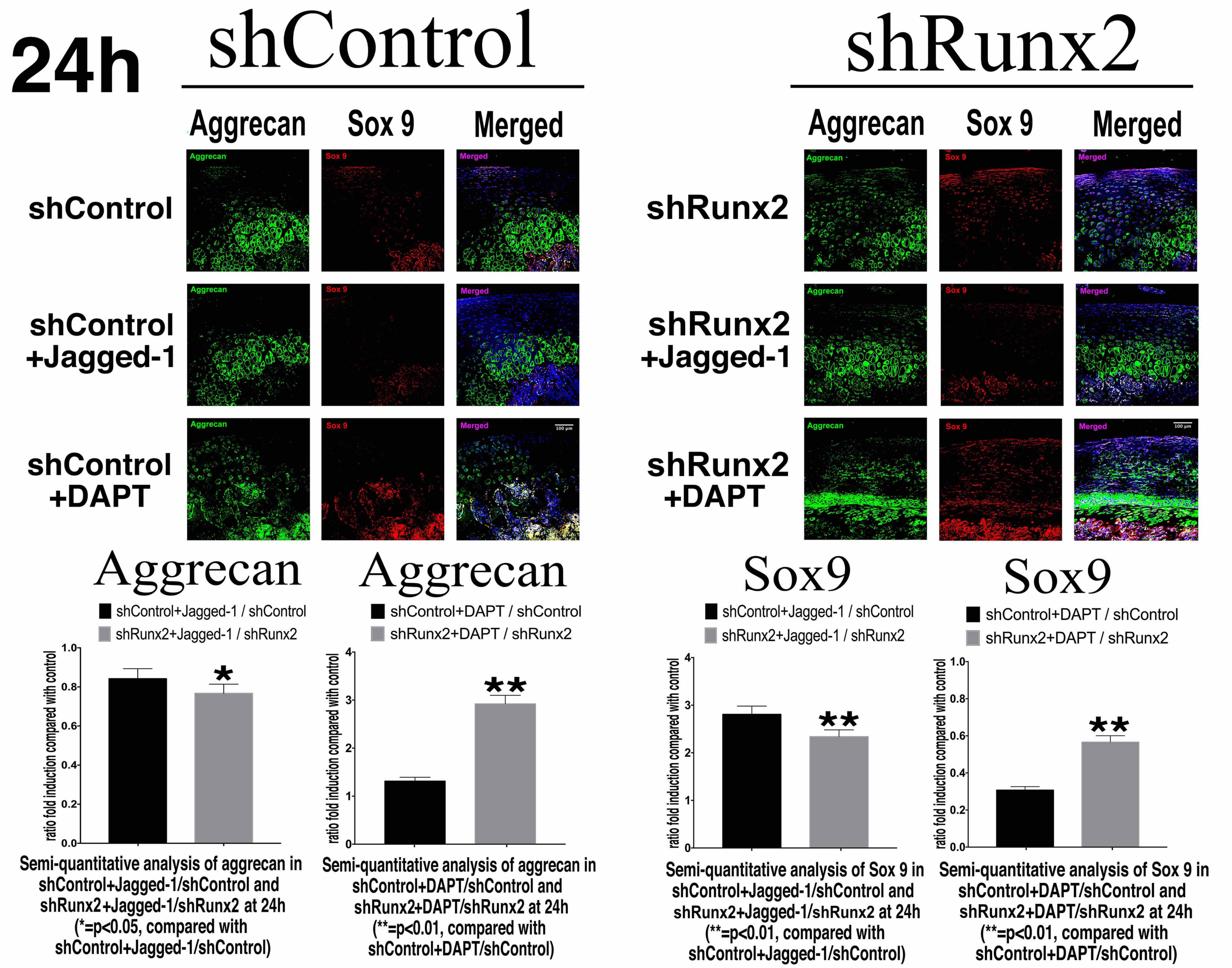

Supplemental Figure 9:
Immunofluorescent staining and semi-quantifications on the change rates of aggrecan and Sox 9 positive cells after Jagged-1 and DAPT treatments. (green=aggrecan, red=Sox 9)

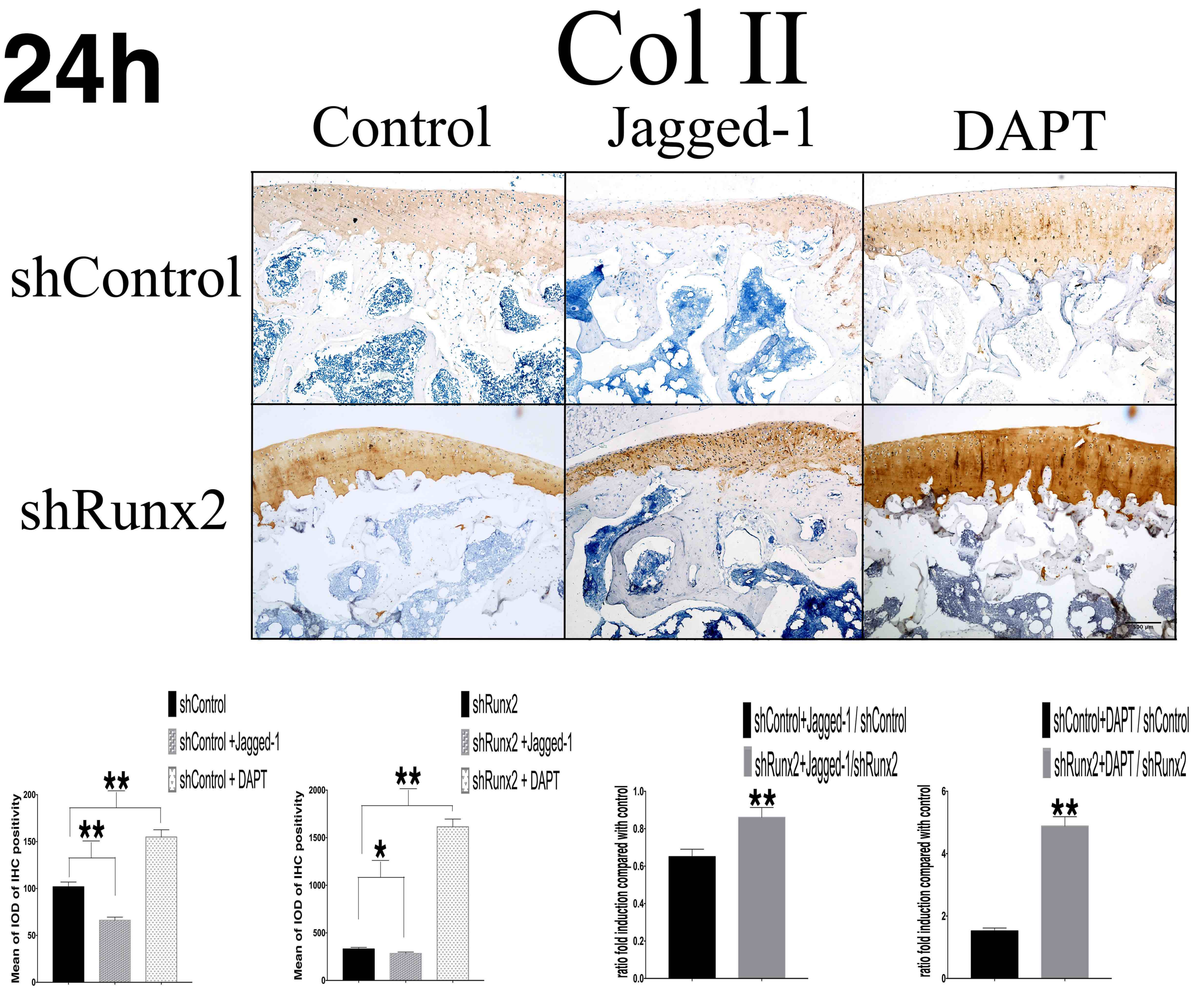

Semi-quantitative analysis of Col II immuno-histochemical staining (shControl as control, **=p<0.01, IOD: integrated optical density)

Semi-quantitative analysis of Col II immuno-histochemical staining (shRunx2 as control, *=p<0.05,**=p<0.01, IOD: integrated optical density)

Semi-quantitative analysis of Col II in shControl+Jagged-1/shControl and shRunx2+Jagged-1/shRunx2 at 24h (**=p<0.01, compared with shControl+Jagged-1/shControl)

Semi-quantitative analysis of Col II in shControl+DAPT/shControl and shRunx2+DAPT/shRunx2 at 24h (**=p<0.01, compared with shControl+DAPT/shControl)

Supplemental Figure 10:
Immunohistochemical staining and semi-quantifications of Col II in rat knee articularcartilage after Jagged-1 and DAPT treatments.

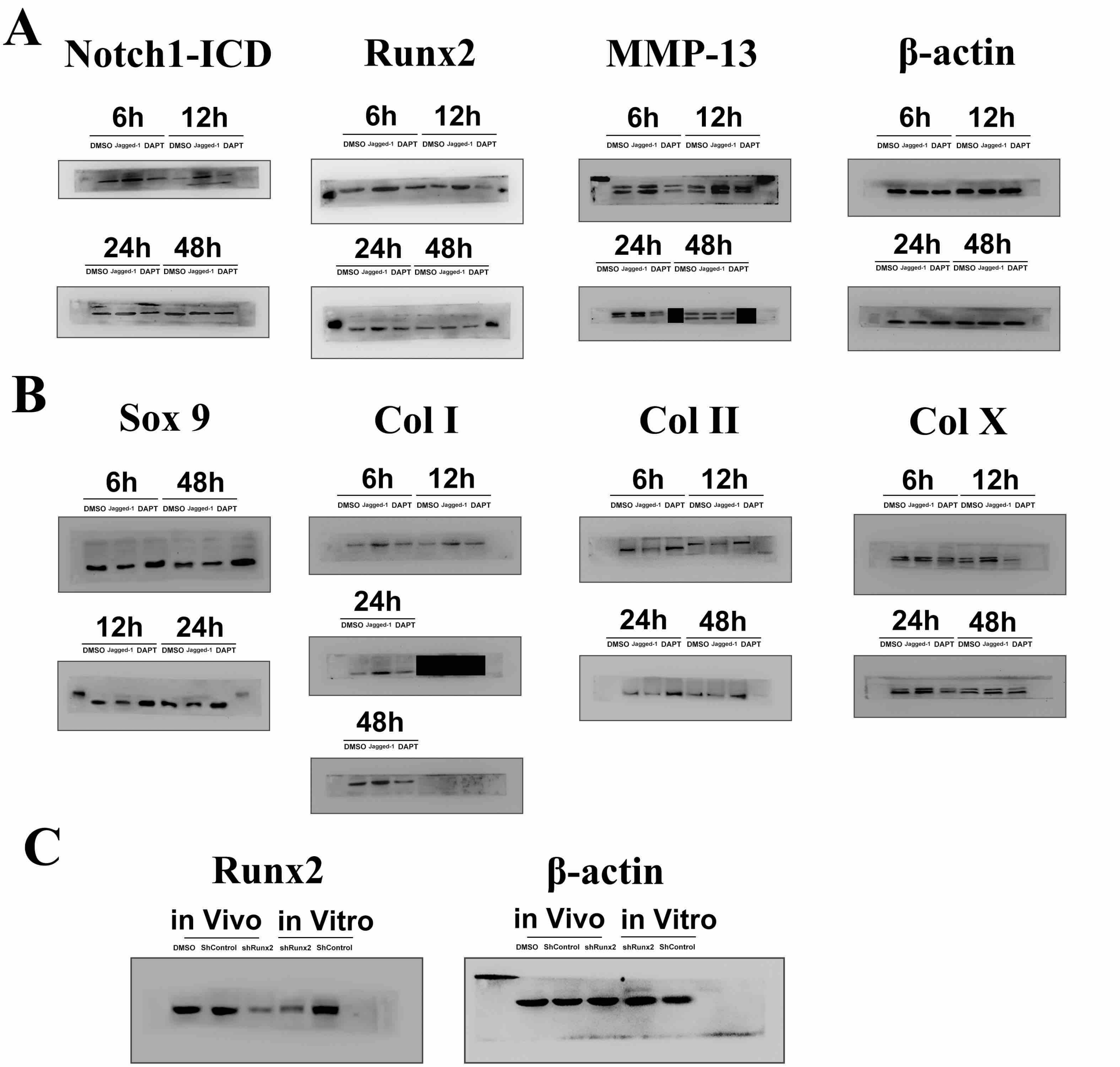

Supplemental Figure 11:
Full images of immunoblots for Fig 1 (A), Fig S2 (B) and Fig S4 (C).
